# Overcoming treatment challenges in myelofibrosis and polycythemia vera: the role of ruxolitinib

**DOI:** 10.1007/s00280-016-3012-z

**Published:** 2016-03-26

**Authors:** Jeffrey C. Bryan, Srdan Verstovsek

**Affiliations:** Pharmacy Clinical Programs, Division of Pharmacy, The University of Texas MD Anderson Cancer Center, 1515 Holcombe Boulevard, Houston, TX 77030 USA; Division of Cancer Medicine, Department of Leukemia, The University of Texas MD Anderson Cancer Center, Houston, TX USA

**Keywords:** JAK inhibition, Myelofibrosis, Myeloproliferative neoplasm, Polycythemia vera, Ruxolitinib

## Abstract

Myelofibrosis (MF) and polycythemia vera (PV) are *BCR*-*ABL1*-negative myeloproliferative neoplasms associated with somatic hematopoietic stem cell mutations leading to over activation of JAK–STAT signaling. MF and PV are pathogenically related and share specific clinical features such as splenomegaly and constitutional symptoms. The MF phenotype is dominated by the effects of progressive bone marrow fibrosis resulting in shortened survival. In contrast, elevated thrombosis risk due to erythrocytosis is the primary clinical concern in PV. Ruxolitinib, an oral JAK1/JAK2 inhibitor, is approved in the USA for the treatment of patients with intermediate- or high-risk MF and patients with PV who have had an inadequate response to or are intolerant of hydroxyurea. For MF, results of two phase III studies demonstrated that ruxolitinib therapy reduced spleen volume and MF-related symptom burden, improved quality-of-life measures, and was associated with prolonged overall survival. Treatment benefits were generally sustained with continued therapy. Dose-dependent cytopenias were common but generally manageable with transfusions (for anemia), dose reduction, or treatment interruption. Optimal dosing management is critical to maintain long-term treatment benefit, because cessation of therapy resulted in rapid return of symptoms to baseline levels. Results of the phase III PV trial showed that ruxolitinib was significantly more effective than standard therapy in controlling hematocrit levels and improving splenomegaly and PV-related symptoms. Only 1 of 110 patients in the ruxolitinib arm compared with 6 of 112 patients in the control arm experienced a thromboembolic event through week 32. Grade ≥3 cytopenias were uncommon.

## Introduction

Philadelphia chromosome–negative myeloproliferative neoplasms (MPNs), including myelofibrosis (MF), polycythemia vera (PV), and essential thrombocythemia (ET), are genetically related, but heterogeneous chronic diseases characterized by overactive signaling through the Janus kinase (JAK)-signal transducer and activator of transcription (STAT) pathway as the central pathogenic mechanism [1–5]. The JAK–STAT pathway plays an essential role in normal hematopoiesis by mediating incoming signals from hematopoietic growth factors such as erythropoietin and thrombopoietin in hematopoietic stem cells [6]. The term “myelofibrosis” refers collectively to patients with de novo, primary myelofibrosis (PMF) and those with myelofibrotic transformation from PV to post-PV MF or from ET to post-ET MF [7, 8].

**Fig. 1:**
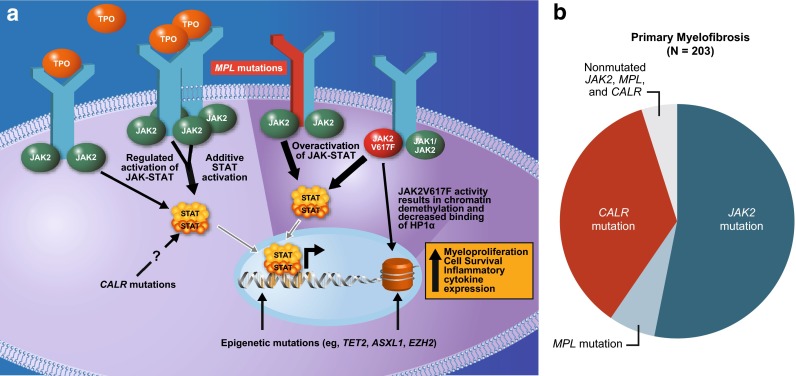
**a** Mutations underlying the pathobiology of myelofibrosis. Mutations commonly affect the JAK–STAT pathway, including those that occur directly in *JAK2* such as the *JAK2*V617F mutation, or indirectly such as those in *MPL*, which encodes the thrombopoietin receptor, or in *CALR*, which may activate STATs through an unknown mechanism. Somatic mutations in epigenetic modifiers can also result in increased myeloproliferation, survival, and cytokine expression in myelofibrosis. From [12]. Copyright © 2014. **b** The approximate proportion of patients with *JAK2*, *MPL*, and *CALR* mutations was examined in 203 patients with primary myelofibrosis. From [10].Copyright © 2013 Massachusetts Medical Society. Reprinted with permission from Massachusetts Medical Society. *CALR* calreticulin, *JAK* Janus kinase, *MPL* myeloproliferative leukemia virus oncogene, STAT signal transducer and activator of transcription

Although our understanding of the relationship between MPN genotype and phenotype remains incomplete, tremendous progress has been made in recent years in elucidating the complexity of somatic mutations that drive MPN pathogenesis and influence patients’ prognoses. Prompted by the discovery of the *JAK2*V617F gain-of-function mutation in more than 50 % of patients with PMF or ET and at least 95 % of patients with PV [4], the importance of JAK–STAT pathway dysregulation in the pathogenesis of MPNs is now well established [5]. In addition to the *JAK2*V617F mutation, which promotes constitutive activation of JAK2, other somatic stem cell mutations have been identified in patients with MPNs that lead to over activation of JAK–STAT signaling, including mutations in the thrombopoietin receptor gene (*MPL*) [4, 5, 9] and the calreticulin gene (*CALR*) [5, 10, 11] (Fig. 1 [10, 12]). In the vast majority of patients, *JAK2*, *MPL*, and *CALR* mutations appear to be mutually exclusive and are considered principal drivers of neoplastic myeloproliferation [10, 11]. However, the diagnostic value of these mutations is limited, as none is specific for any one type of MPN and a small minority of patients with MPNs lack *JAK2*, *MPL*, and *CALR* mutations [13, 14]. Furthermore, patients with MPNs often carry multiple additional mutations that may affect clinical phenotype and/or prognosis [5, 14, 15].

In addition to its essential role in hematopoiesis, the JAK–STAT pathway is central to cytokine activation and signaling in the immune system [6]. It is well documented that patients with MF have abnormally high levels of circulating inflammatory cytokines, including tumor necrosis factor alpha (TNF-α) and interleukin (IL)-6 [16], which appear to be fueled by aberrant cytokine secretion of both malignant and nonmalignant cells in the bone marrow [17]. Moreover, JAK1 hyperactivity has been noted in patients with MF [18] and may be due to cytokine hyperstimulation. It is believed that abnormally high levels of circulating inflammatory cytokines are a major cause for the burden of constitutional symptoms in patients with MPNs [16, 19].

Ruxolitinib, an orally bioavailable inhibitor of JAK1 and JAK2, is currently the only pharmacotherapy with approved indications in MF, and it has been recently approved by the US Food and Drug Administration for the treatment of patients with PV with an inadequate response to or intolerant of hydroxyurea. Ruxolitinib oral tablets are available in strengths of 5, 10, 15, 20, and 25 mg, allowing for individualized dosing regimens (per dosing recommendations in the prescribing information [20]). In this review, we summarize the efficacy and safety data for ruxolitinib in both indications and discuss specific pharmacologic properties relevant for its safe and effective administration.

## Ruxolitinib: general pharmacology

Ruxolitinib is an equipotent inhibitor of JAK1 [mean half maximal inhibitory concentration (IC_50_) = 3.3 nM] and JAK2 (mean IC_50_ = 2.8 nM) in vitro, with at least 100-fold less inhibitory activity against JAK3 [18]. Early results obtained with a mouse model of *JAK2*V617F-induced MPN showed that ruxolitinib was highly effective in reducing splenomegaly and lowering IL-6 and TNF-α levels and prolonged survival. The effects of ruxolitinib on circulating cytokine levels were subsequently confirmed in phase II and III clinical studies and are believed to be integral to the efficacy of ruxolitinib [16, 21].

Ruxolitinib has a terminal half-life of approximately 3 h [22] and is predominantly metabolized by CYP3A4 and to a lesser extent by CYP2C9, with 74 and 22 % of a ruxolitinib dose recovered in urine and feces, respectively, within 24 h of oral administration in healthy human volunteers (<1 % was unchanged drug) [20, 23]. Ruxolitinib plasma exposure [area under the curve from time 0 extrapolated to infinite time (AUC_0–inf_)] increased by 91 and 27 % following concomitant administration of ketoconazole (a potent CYP3A4 inhibitor) and erythromycin (a moderate CYP3A4 inhibitor), respectively [24]. Pretreatment with the potent CYP3A4 inducer rifampin resulted in a 71 % decrease in ruxolitinib AUC_0–inf_, but had a limited impact on the overall pharmacodynamic activity, which decreased by 10 % [24]. This may be explained in part by an increase in the relative abundance of active ruxolitinib metabolites with rifampin coadministration [24].

## Myelofibrosis

### Natural history and prognosis

**Fig. 2:**
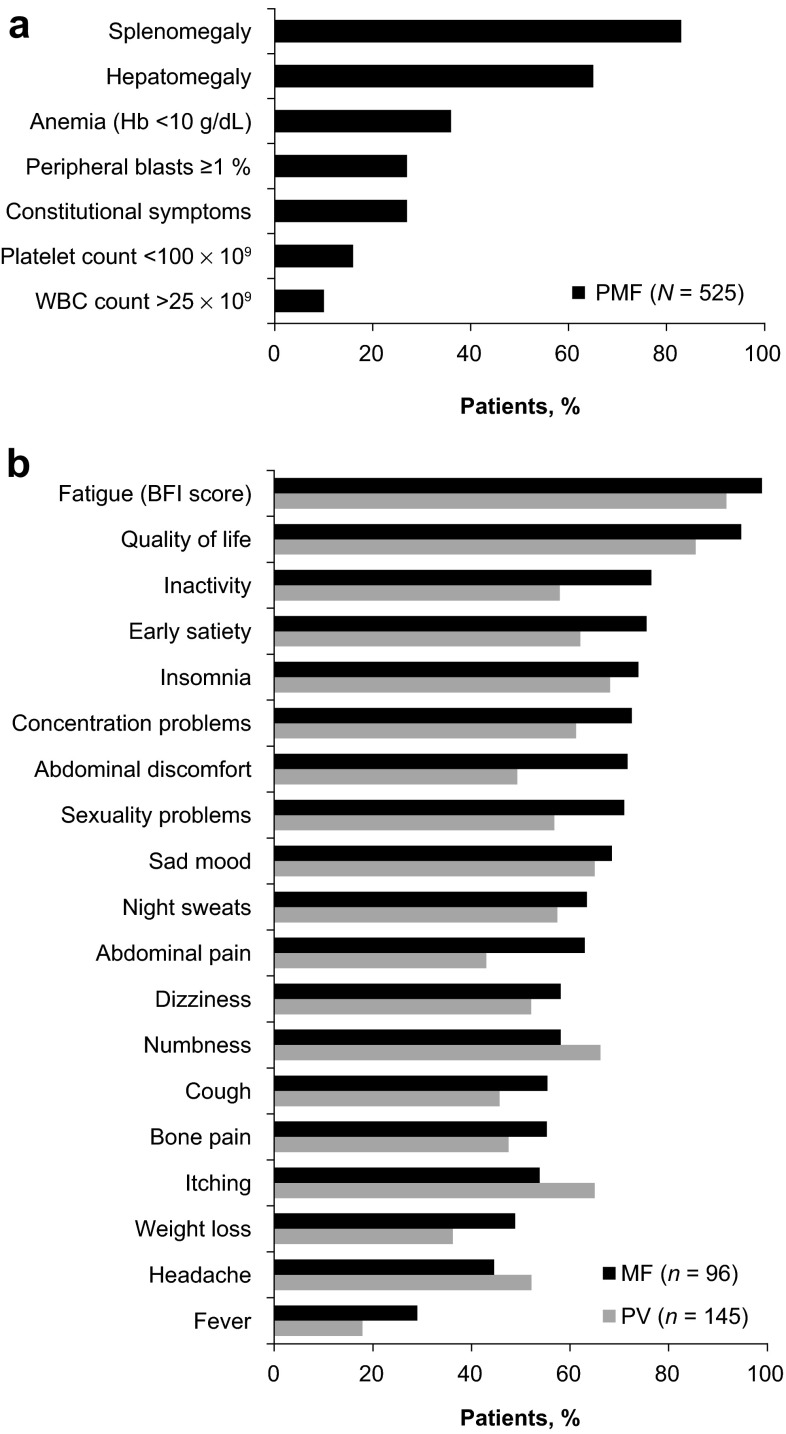
**a** Prevalence of disease characteristics at diagnosis of 525 patients with primary myelofibrosis examined by the International Working Group for Myeloproliferative Neoplasms Research and Treatment [34]. **b** Prevalence of common symptoms per the Myeloproliferative Neoplasm Symptom Assessment Form among 96 patients with myelofibrosis and 145 patients with polycythemia vera [31]. *BFI* Brief Fatigue Inventory, *Hb* hemoglobin, *MF* myelofibrosis, *PMF* primary myelofibrosis, *PV* polycythemia vera, *WBC* white blood cell

MF is a chronic disease marked by progressive bone marrow fibrosis, ineffective erythropoiesis, excess production of dysplastic megakaryocytes, extramedullary hematopoiesis, systemic inflammation with excess circulating levels of proinflammatory cytokines, cachexia, and shortened survival [16, 25–27]. The development and progression of fibrosis in the marrow is likely a reaction by stromal cells to the malignant hematopoietic stem cell clones and eventually leads to cytopenias (ineffective hematopoiesis) and/or to extramedullary hematopoiesis as compensation for the diminished capacity of the bone marrow [28, 29]. The main clinical manifestations of MF include splenomegaly and a variety of troublesome symptoms (Fig. 2) [25, 26, 30, 31], which are a major source of morbidity and poor quality of life (QoL) [32–35]. Common symptoms include abdominal discomfort, early satiety, itching, bone pain, muscle pain, fatigue, dyspnea, and insomnia [31, 35]. Many patients also experience anemia, which may necessitate red blood cell transfusions [25, 32–34], and hepatomegaly is common among patients who underwent palliative splenectomy [36–38]. Patients with MF have an increased risk of developing secondary acute myeloid leukemia [39]. Leukemic transformation is thought to result from the accumulation of deleterious genetic events in addition to the mutations affecting JAK–STAT signaling, but the precise contribution of individually acquired mutations to the process of transformation is not well understood [40].

Among patients with MPNs, those with PMF have the worst prognosis, with a median life expectancy of 6 years at the time of diagnosis [14]. Patients with MF may die from a variety of complications related to disease progression [32, 41]. Risk factors for shortened survival that have been validated in various prognostic models include age >65 years, constitutional symptoms (fever, night sweats, weight loss), hemoglobin <10 g/dL, leukocytes >25 × 10^9^/L, circulating blasts ≥1 % [32, 34], unfavorable karyotype, platelets <100 × 10^9^/L, and the need for red blood cell transfusions [33]. Median survival varies from approximately 11 years for those with low-risk disease to 2 years for those with high-risk disease [32]. Additional variables that have demonstrated prognostic value outside of these models include mutations associated with worse (*ASXL1*) or better (*CALR*) prognosis [15, 42, 43], the number of mutations present [43], and the levels of circulating cytokines [44]. Current prognostic models also do not consider the potential impact of splenomegaly, fibrosis grade, low cholesterol, or comorbidities on survival [45–49].

### Limitations of traditional therapies

Allogeneic hematopoietic stem cell transplantation is the only potentially curative option for MF and provides resolution of bone marrow fibrosis in the majority of successful transplants [26, 50, 51]. However, because of the high risks of treatment-related morbidity and mortality, allogeneic hematopoietic stem cell transplantation is generally limited to younger patients with a survival expectancy of <5 years, with consideration of the patient’s overall benefit-risk profile [26, 52]. Before the development of targeted inhibitors, conventional therapies addressing specific signs or symptoms of MF were used in a multimodal approach essentially intended to provide palliation. Treatment with immunomodulatory agents such as thalidomide, lenalidomide, or pomalidomide may benefit some patients with MF and anemia; however, recent studies suggest that these agents often have modest activity and/or provide limited long-term benefit with no or marginal effects on splenomegaly [53–57]. Chemotherapies such as hydroxyurea, melphalan, busulfan, and cladribine have been used mainly for the treatment of symptomatic splenomegaly; however, their efficacy was also modest and their use was associated with an increased burden of adverse events [58]. Importantly, conventional therapies have not demonstrated alleviation of constitutional symptoms such as fatigue [35] and have not been shown to result in improved overall survival or disease modification.

For patients with symptomatic splenomegaly who are intolerant of or refractory to pharmacotherapy, splenic irradiation and splenectomy are alternative palliative treatment options. However, both options have significant limitations. Palliative cytoreductive radiotherapy to the spleen, liver, or other sites of extramedullary hematopoiesis often provides nondurable responses and may cause or exacerbate cytopenias [59]. Splenectomy is associated with increased risk of complications and poor postoperative prognosis [37, 60]. Therefore, splenectomy should be considered only for patients who have splenomegaly associated with portal hypertension, severe cytopenias, or other severe symptom, and who have no other treatment options.

## Ruxolitinib for myelofibrosis

In November 2011, ruxolitinib was approved in the USA for the treatment of patients with intermediate- or high-risk MF [20]. Outside the USA, ruxolitinib is approved for the treatment of MF-related splenomegaly and symptoms in more than 80 countries. The initial approval of ruxolitinib was based on the results of two pivotal phase III clinical trials, COntrolled MyeloFibrosis study with ORal JAK inhibitor Treatment (COMFORT)-I [21] and -II [61].

### Efficacy

Patients with intermediate-2 or high-risk MF, platelet counts ≥100 × 10^9^/L, and splenomegaly received ruxolitinib or placebo in a randomized double-blind study (COMFORT-I), or ruxolitinib or best available therapy (BAT, most commonly hydroxyurea, 47 % of patients) in a randomized open-label study (COMFORT-II) [21, 61]. Based on assessments by magnetic resonance imaging or computed tomography, the primary endpoint, a ≥35 % reduction in total spleen volume (from baseline to week 24 for COMFORT-I and week 48 for COMFORT-II), was achieved by 41.9 % of patients in the ruxolitinib group versus 0.7 % in the placebo group in COMFORT-I and in 28 % in the ruxolitinib group versus 0 % in the BAT group in COMFORT-II [21, 61]. Overall, 97 % of patients in both studies experienced some degree of reduction in spleen volume upon treatment with ruxolitinib [21, 61]. Long-term ruxolitinib therapy was associated with marked and durable reductions in splenomegaly. In COMFORT-I, the median reduction from baseline in spleen volume was 34.9 % at week 96 and 34.1 % at week 144 in patients randomized to ruxolitinib [41, 62]. Patients in COMFORT-II who achieved a ≥35 % reduction in spleen volume had a 50 % probability of maintaining this level of improvement at week 144 [63]. At the time of the 3-year analyses, 49.7 and 45 % of patients randomized to ruxolitinib in COMFORT-I and COMFORT-II, respectively, were still being treated [41, 62, 63].

**Fig. 3:**
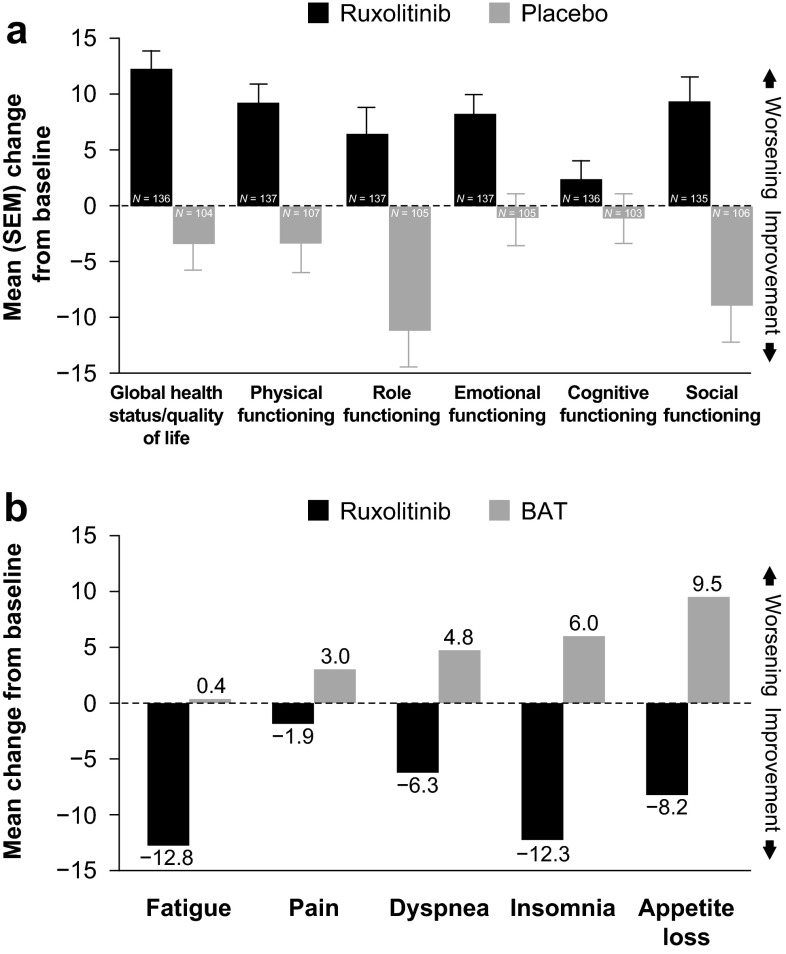
**a** Mean change from baseline to week 24 in EORTC-QLQ-C30 global health status and functional scales results in COMFORT-I . From [21]. Copyright © 2012 Massachusetts Medical Society. Reprinted with permission from Massachusetts Medical Society**b** Mean changes from baseline to week 48 in EORTC-QLQ-C30 symptom scores in COMFORT-II. *EORTC*-*QLQ*-*C30* European Organization for Research and Treatment of Cancer Quality-of-Life questionnaire core model 30 . From [61]. Copyright © 2012 Massachusetts Medical Society. Reprinted with permission from Massachusetts Medical Society.

An important secondary objective of the COMFORT trials was the assessment of changes in symptom burden, which is poorly addressed with traditional therapies. In COMFORT-I, 45.9 % of patients who received ruxolitinib versus 5.3 % of those who received placebo (*P* < 0.001) achieved a ≥50 % improvement in total symptom score at week 24, as assessed by the modified Myelofibrosis Symptom Assessment Form version 2 [21]. Marked improvements in role functioning and QoL per the European Organization for Research and Treatment of Cancer (EORTC) Quality-of-Life questionnaire core model (QLQ-C30) were also noted in ruxolitinib-treated patients (Fig. 3) [21, 64]. Similarly, in COMFORT-II, patients treated with ruxolitinib experienced mean improvements in fatigue, pain, dyspnea, insomnia, and appetite loss at week 48 per their responses on the EORTC-QLQ-C30, whereas patients treated with BAT experienced mean worsening of those symptoms (Fig. 3) [61]. Some of the reduction in symptom burden may be due to a decrease in the proinflammatory state, because ruxolitinib has been shown to decrease cytokine levels in patients with MF, which coincided with improvement in symptoms and splenomegaly [16, 65, 66]. Ruxolitinib-mediated symptom improvement and QoL benefits were durable among patients remaining on therapy based on recent 2- and 3-year follow-up data from the COMFORT studies [41, 62, 63].

In both COMFORT studies, efficacy was not dependent on the presence of the *JAK2*V617F mutation [21, 61]. Moreover, the rapid reductions in splenomegaly and symptom burden seen with ruxolitinib were not reflected by corresponding changes in allele burden, which generally were modest at the time of the primary analyses of COMFORT-I and COMFORT-II [21, 61, 63]. The absence of *JAK2*V617F should not preclude treatment with ruxolitinib and change in *JAK2*V617F allele burden is not useful as an indicator or predictor of short-time treatment success or as a surrogate measure of spleen size or symptom burden reduction.

### Effect on biomarkers

A hallmark of advanced MF is the preponderance of cachexia and systemic inflammation, manifested by unwanted weight loss, hypocholesterolemia, low-grade fever, fatigue, and other constitutional symptoms [31, 45, 67]. In addition, MF is characterized by increased plasma levels of proinflammatory cytokines such as IL-6, IL-8, TNF-α, and lipocalin-2 [16, 44, 68]. Some of these cytokines, such as lipocalin-2 and IL-8, have been implicated directly in MF pathogenesis. Increased IL-8 expression in megakaryocytes derived from malignant stem cells may contribute to excess fibroblast proliferation in the bone marrow [69], whereas lipocalin-2 may promote oxidative damage of normal but not malignant CD34+ cells as well as osteoclastogenesis and fibrosis [68]. IL-6 is recognized as a mediator of cancer-related cachexia [70] and thus may be a driver of cachexia in MF.

Results from clinical studies of ruxolitinib in MF demonstrated that ruxolitinib not only mitigated cachexia but also had a positive effect on various markers of inflammation. In the phase 1/2 study of ruxolitinib in MF, ruxolitinib promoted rapid reductions in plasma levels of a large number of inflammatory markers, including but not limited to IL-6, TNF-α, and C-reactive protein, an acute-phase marker of inflammation [16]. In COMFORT-I, ruxolitinib versus placebo was associated with significant improvements in weight (mean weight gain of 3.9 kg with ruxolitinib vs mean weight loss of 1.9 kg with placebo, *P* < 0.0001) and metabolic status (e.g., mean increase in total cholesterol of 26.4 % vs mean decrease in 3.3 %, *P* < 0.0001) at 24 weeks, and sustained improvement was observed with long-term therapy [71]. These metabolic improvements were accompanied by median reductions in plasma levels of close to 40 % for IL-6 and TNF-α and more than 70 % for C-reactive protein at week 24 [21]. In contrast, inflammation markers showed no (TNF-α) or marginal improvement (IL-6, C-reactive protein) with placebo. Furthermore, median plasma leptin levels increased approximately twofold with ruxolitinib and decreased slightly with placebo [21].

Results from animal models of MF suggest that increased production of proinflammatory cytokines originates not only from malignant cells but also from nonmalignant cells and is rapidly and potently inhibited by ruxolitinib [17]. Of note, deletion of STAT3 in mutant clones has been shown to be insufficient to suppress inflammatory signaling [17]. This finding supports the importance of inhibiting JAK–STAT signaling in nonmalignant cells to mitigate inflammation-related symptoms and would explain the effectiveness of ruxolitinib in providing rapid symptom mitigation without clinically significant reduction in mutant allele burden.

### Survival and disease modification

Overall, data from the COMFORT studies suggest that ruxolitinib was associated with improved overall survival compared with placebo or BAT in patients with MF [21, 41, 61, 72, 73]. Kaplan–Meier analyses of overall survival based on the intention-to-treat principle showed hazard ratios in favor of ruxolitinib versus placebo or BAT despite a potential bias in favor of the control arms caused by patient crossover to ruxolitinib [21, 61–63, 74]. In COMFORT-II, the survival advantage observed was independent of the mutation profile, including prognostically detrimental mutations [75]. Furthermore, an ad hoc comparison of COMFORT-II data with those from a historical control group determined that patients with PMF receiving ruxolitinib had longer overall survival from the time of diagnosis than patients receiving conventional therapy [median survival 5 vs 3.5 years; hazard ratio (95 % CI) 0.61 (0.41–0.91), *P* = 0.0148], suggesting that ruxolitinib may modify the natural history of PMF [76]. This hypothesis is supported by long-term follow-up data from the phase II and III trials of ruxolitinib in MF showing treatment-associated changes in bone marrow fibrosis and/or allele burden. Comparative analyses of bone marrow biopsies from patients enrolled in the ruxolitinib phase II study and a matched cohort treated with BAT showed that the proportions of patients who had improved or stabilized bone marrow fibrosis after 1–5 years of treatment was greater with ruxolitinib than BAT [77]. Conversely, the odds of worsening fibrosis were lower with ruxolitinib versus BAT [odds ratio at 5 years (95 % CI) 0.07 (0.01–0.34)] [77]. A recent post hoc analysis from the COMFORT-I study further showed that long-term therapy with ruxolitinib resulted in partial or complete molecular remission of *JAK2*V617F allele burden in 20 and six patients, respectively [78]. Of note, complete resolution of bone marrow fibrosis documented in two case reports of patients with post-PV MF appeared to be accompanied by complete molecular remission [79, 80]. Together, these findings suggest that long-term therapy can result in profound disease modification, including sustained remission of bone marrow fibrosis and malignant clonal burden. Nevertheless, the general prolongation of survival observed with ruxolitinib versus placebo or BAT in the COMFORT trials may be the result of overall improvement in patients’ health status, including the reduction of cachexia (unwanted weight loss) and other constitutional symptoms, which are known prognostic factors in MF [32].

### Safety and tolerability

The most common nonhematologic adverse reactions occurring in a greater proportion of patients receiving ruxolitinib versus placebo were ecchymosis, dizziness, and headache (mostly grade 1 or 2), whereas abdominal pain and fatigue, which are typical disease-related symptoms, occurred less frequently in the ruxolitinib arm versus the placebo arm (Table 1) [21]. In COMFORT-II, the most common nonhematologic adverse events occurring in more patients treated with ruxolitinib versus BAT were diarrhea (23 vs 12 %) and asthenia (18 vs 10 %); most nonhematologic adverse events were grade 1 or 2 [61].

**Table 1:** Nonhematologic adverse events in ≥10 % of patients who received ruxolitinib in COMFORT-I From [21]. Copyright © 2012 Massachusetts Medical Society. Reprinted with permission from Massachusetts Medical Society

Percent of patients	Ruxolitinib (*n* = 155)		Placebo (*n* = 151)	
	All grades	Grade 3 or 4	All grades	Grade 3 or 4
Fatigue	25.2	5.2	33.8	6.6
Diarrhea	23.2	1.9	21.2	0
Peripheral edema	18.7	0	22.5	1.3
Ecchymosis	18.7	0	9.3	0
Dyspnea	17.4	1.3	17.2	4.0
Dizziness	14.8	0.6	6.6	0
Nausea	14.8	0	19.2	0.7
Headache	14.8	0	5.3	0
Constipation	12.9	0	11.9	0
Vomiting	12.3	0.6	9.9	0.7
Pain in extremity	12.3	1.3	9.9	0
Insomnia	11.6	0	9.9	0
Arthralgia	11.0	1.9	8.6	0.7
Pyrexia	11.0	0.6	7.3	0.7
Abdominal pain	10.3	2.6	41.1	11.3

Ruxolitinib inhibits normal erythropoietin and thrombopoietin signaling through JAK2 (through the erythropoietin and thrombopoietin receptors, respectively) [6], often resulting in dose-dependent anemia and thrombocytopenia [21, 61]. Indeed, in the COMFORT studies, the most common hematologic adverse reactions with ruxolitinib were thrombocytopenia and anemia, some of which were grade 3 or 4 (Table 2) [21, 61]. However, of 301 patients randomized to ruxolitinib treatment in the two trials, only 1 patient discontinued ruxolitinib for anemia and 2 patients discontinued ruxolitinib for thrombocytopenia. Instead, cytopenias were managed by dose modifications and treatment interruptions or with red blood cell transfusions for anemia [21, 61]. In COMFORT-I, 77 % of patients with a baseline platelet count of 100–200 × 10^9^/L and 39 % of patients with a baseline platelet count >200 × 10^9^/L were receiving a lower dose at week 24 than at baseline [81]. Overall the incidence of adverse events decreased over time, with most occurring during the first 12 weeks of treatment [21, 41, 61, 63]. In both trials, mean hemoglobin levels decreased initially, but recovered after the first 8–12 weeks to new steady-state levels slightly lower than baseline [21, 61]. Similarly, ruxolitinib-treated patients initially required more blood transfusions than placebo-treated patients, but transfusion rates gradually returned close to baseline values when assessed over a 36-week period [21]. In COMFORT-I, patients treated with ruxolitinib who experienced grade 3 or 4 anemia had similar improvements in symptoms as those who did not have anemia [21]. Mandatory dose reductions for thrombocytopenia occurred mostly during the first 8–12 weeks of treatment when mean platelet count decreased; after this period, a stabilization of mean platelet counts was observed [82].

**Table 2:** Hematologic adverse events^a^ in the phase III COMFORT trials, regardless of relation to study drug [21, 61]

Percent of patients with any grade event (percent with grade 3 or 4 event)	COMFORT-I		COMFORT-II	
	Ruxolitinib (*n* = 155)	Placebo (*n* = 151)	Ruxolitinib (*n* = 146)	BAT (*n* = 73)
Anemia	96.1 (45.2)	86.8 (19.2)	96 (42)	94 (31)
Thrombocytopenia	69.7 (12.9)	30.5 (1.3)	68 (8)	29 (7)
Neutropenia	18.7 (7.1)	4.0 (2.0)	NR	NR

*BAT* best available therapy, *COMFORT* COntrolled MyeloFibrosis study with ORal JAK inhibitor Treatment, *NR* not reported

^a^New or worsening hematologic events based on laboratory values

In COMFORT-I, at the 3-year follow-up, four patients originally randomized to ruxolitinib and four patients randomized to placebo experienced disease progression to secondary acute myeloid leukemia [21, 41, 62]. In COMFORT-II, at the 3-year follow-up, five patients (3.4 %) in the ruxolitinib arm and four patients (5.5 %) in the BAT arm experienced leukemic transformation [73].

Although rare adverse events of fever, respiratory distress, hypotension, and multi-organ failure have been reported after treatment discontinuation [20], experience from the placebo-controlled COMFORT-I study provided no evidence that treatment discontinuation per se was associated with serious adverse events [21, 41]. If a patient experiences one of these adverse events after the drug has been withdrawn or while tapering the dose, the intercurrent illness should be evaluate and treated, and restarting or increasing the dose of ruxolitinib should be considered [20]. If a patient needs to discontinue the use of ruxolitinib for a reason other than cytopenia, a gradual tapering of the dose by 5 mg twice daily each week may be considered to reduce the severity of returning symptoms [20]. Furthermore, the use of corticosteroids following discontinuation of ruxolitinib may be considered in specific cases where tapering of ruxolitinib is not possible (e.g., in cases of severe thrombocytopenia requiring immediate treatment discontinuation) and abrupt ruxolitinib withdrawal results in an acute return of systemic inflammatory symptoms.

### Dose management to maximize efficacy and minimize treatment-related cytopenias

The recommended starting and maintenance dose of ruxolitinib for the treatment of patients with MF is dependent on the baseline platelet count (Table 3) [20]. If response is insufficient, the dose may be increased beginning 4 weeks after initiation of therapy to a maximum of 25 mg twice daily for patients with starting platelet counts ≥100 × 10^9^/L and to a maximum of 10 mg twice daily for patients with starting platelet counts of 50 to <100 × 10^9^/L [82]. These increases may be undertaken in patients who fail to achieve a ≥50 % reduction in palpable spleen length or a ≥35 % reduction in spleen volume, as long as adequate platelet and absolute neutrophil counts are maintained [20]. Once-daily doses are not as effective as twice-daily dosing and should not be used unless specifically indicated [20]. If a dose is missed, the patient should take the next scheduled dose at the regular time [20]. Dose modifications should not be implemented on the basis of allele burden, because, as discussed above, allele burden reduction is not a clinically validated outcome marker in MF.

**Table 3:** Ruxolitinib regular starting doses and starting doses for patients with concomitant use of strong CYP3A4 inhibitors or fluconazole [20]

Population	Platelet count	Ruxolitinib starting dose
Myelofibrosis		
Regular starting doses based on platelet count	>200 × 10^9^/L	20 mg twice daily
	100 × 10^9^ to 200 × 10^9^/L	15 mg twice daily
	50 × 10^9^ to <100 × 10^9^/L	5 mg twice daily
	<50 × 10^9^/L	Not recommended
Concomitant use of strong CYP3A4 inhibitors or fluconazole ≤200 mg	≥100 × 10^9^ to 200 × 10^9^/L	10 mg twice daily
	50 × 10^9^ to <100 × 10^9^/L	5 mg twice daily
Fluconazole >200 mg	Any	Concomitant use not recommended
Polycythemia vera		
Regular starting dose	Any	10 mg twice daily
Concomitant use of strong CYP3A4 inhibitors or fluconazole ≤200 mg	Any	5 mg twice daily
Fluconazole >200 mg	Any	Concomitant use not recommended

Experience from the COMFORT studies showed that careful monitoring of complete blood counts and adjustment of the dosing regimen accordingly are key to long-term therapeutic success in patients with MF, as adherence to therapy and avoidance of extended interruptions are important factors in maintaining response to therapy [21, 61]. If ruxolitinib needs to be stopped, symptoms can be expected to return within 1 week, usually to pretreatment levels [20]. Therefore, timely management of cytopenias with dose reductions, particularly during the first 3 months of therapy, is generally preferable to the risk of extended treatment interruptions that may reverse treatment gains. Prior to initiating ruxolitinib, a complete blood count must be performed, with subsequent monitoring during therapy every 2–4 weeks until doses stabilize, and then as clinically indicated [20, 82]. For patients with a starting platelet count of ≥100 × 10^9^/L, if at any point the platelet count falls below 50 × 10^9^/L, treatment should be interrupted until the count recovers; for patients with a baseline platelet count of 50 to <100 × 10^9^/L, treatment should be interrupted if platelet count falls below 25 × 10^9^/L [20]. Frequent monitoring and prompt dose adjustments should be employed to avoid these occurrences. Detailed dosing recommendations for cytopenias can be found in the prescribing information [20, 82].

In patients requiring modifications from the starting dose, clinical evidence suggests that ruxolitinib can be effective long-term at titrated doses as low as 10 mg twice daily [82–84]. In trials of patients with MF with low platelet counts (between 50 and 100 × 10^9^/L), dosing of ruxolitinib was initiated at 5 mg twice daily followed by escalation by 5 mg daily every 4 weeks to 10 mg twice daily or higher in patients with adequate platelet counts [83, 85]. Preliminary findings suggest that this dosing strategy is effective in reducing spleen volume and improving symptoms [83]. Although there are no contraindications for the use of ruxolitinib, treatment is not recommended in patients with baseline platelet counts <50 × 10^9^/L [20].

Patients developing anemia may require blood transfusions and/or dose modifications of ruxolitinib. Patients treated with ruxolitinib in COMFORT-II who also received erythropoietin-stimulating agents (ESA) had a decrease in the rate of grade 3 or 4 anemia within 6 weeks of the first administration of ESA, and administration of ESA did not appear to affect the efficacy of ruxolitinib in reducing spleen volume [86]. Severe neutropenia (absolute neutrophil count <0.5 × 10^9^/L) is generally reversible with interruption of ruxolitinib therapy [81], which may be restarted once absolute neutrophil count recovers [20].

## Polycythemia vera

### Clinical manifestation and prognosis

By definition [87], patients with PV have no or only minor degrees of bone marrow fibrosis, and with a median life expectancy of 13.5 years from the time of diagnosis, their prognosis is much more favorable than that of patients with PMF [14]. However, patients with PV who have some degree of bone marrow fibrosis at diagnosis have a 22 % chance of developing post-PV MF within 10 years, compared with a 7 % chance for patients with no bone marrow fibrosis at diagnosis [88]. Patients with PV typically have high levels of red blood cell mass, and keeping hematocrit levels below 45 % has been shown to be instrumental in minimizing the risk of cardiovascular death and major thrombosis [89]. However, although controlling hematocrit levels is the main concern in patients with PV, the disease is also associated with splenomegaly in approximately 40 % of patients [90], and with a considerable symptom burden, including high prevalence of fatigue (92 %) and pruritus (65 %) reported in a large survey (Fig. 2) [31].

### First-line therapy

Low-dose aspirin is the therapy of choice to reduce the overall risk of vascular events [91], and phlebotomy and/or cytoreductive therapy are commonly used to maintain hematocrit levels of <45 % [89]. Hydroxyurea is the most common first-line cytoreductive therapy for patients at high risk of thrombosis who cannot undergo phlebotomy, require frequent phlebotomy, and/or have splenomegaly or PV-related symptoms [19]. However, some evidence suggests that hydroxyurea is possibly leukemogenic [92] and some patients may not tolerate hydroxyurea or may develop clinical resistance to hydroxyurea [93]. In addition to hydroxyurea, pegylated interferon is frequently used as first-line therapy for patients with PV, based on phase II clinical results that showed high rates of hematologic and molecular responses [94, 95].

## Ruxolitinib for polycythemia vera

In December 2014, ruxolitinib was approved in the USA for the treatment of patients with PV who have had an inadequate response to or are intolerant of hydroxyurea [20]. Approval was based on the demonstration of clinical benefits in phase II and III clinical studies [96, 97].

### Efficacy

In the open-label, multicenter phase III Randomized Study of Efficacy and Safety in Polycythemia Vera with JAK Inhibitor INCB018424 versus Best Supportive Care (RESPONSE), 222 patients with PV and resistance to or intolerance of hydroxyurea were randomized to receive ruxolitinib (starting dose: 10 mg twice daily) or standard therapy. Standard therapy consisted of hydroxyurea (at tolerated doses) in 58.9 %, interferon in 11.6 %, pipobroman in 1.8 %, anagrelide in 7.1 %, immunomodulators (e.g., lenalidomide, thalidomide) in 4.5 %, and no medication in 15.2 % of patients in this treatment arm [98]. Per inclusion criteria, patients had splenomegaly, exhibited phlebotomy dependence (defined as two or more phlebotomies during the 24 weeks before screening and one or more phlebotomies during the 16 weeks before screening), and did not receive prior treatment with a JAK inhibitor. The composite primary endpoint was the proportion of patients with both hematocrit control and a ≥35 % reduction in spleen volume from baseline to week 32. To achieve the endpoint of hematocrit control, a patient had to have ≤1 instance of phlebotomy eligibility during the first 8 weeks after randomization and no instance of phlebotomy eligibility during the remainder of the 32-week study period.

**Fig. 4:**
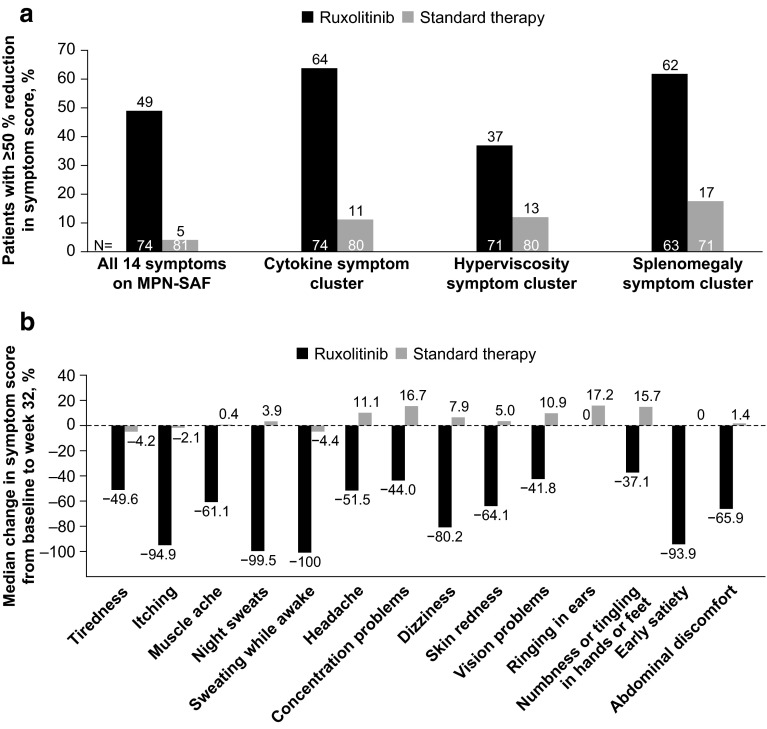
Symptom assessment in the RESPONSE study [98]. **a** The percentage of patients with polycythemia vera who had ≥50 % reduction in the MPN-SAF total symptom score (with regard to 14 symptoms; higher scores indicate greater severity of symptoms) and in total scores for the cytokine symptom cluster (tiredness, itching, muscle ache, night sweats, sweating while awake), the hyperviscosity symptom cluster (vision problems, dizziness, concentration problems, headache, numbness or tingling in the hands or feet, ringing in the ears, skin redness), and the splenomegaly symptom cluster (abdominal discomfort, early satiety) at week 32. **b** The median percentage change from baseline to week 32 in the score for each of the 14 symptoms on the MPN-SAF. Patients with data at both baseline (value >0) and week 32 were included in the analyses for both panels. *Negative values* indicate a reduction in the severity of symptoms. *MPN*-*SAF* Myeloproliferative Neoplasm Symptom Assessment Form From [98]. Copyright © 2015 Massachusetts Medical Society. Reprinted with permission from Massachusetts Medical Society

**Fig. 5:**
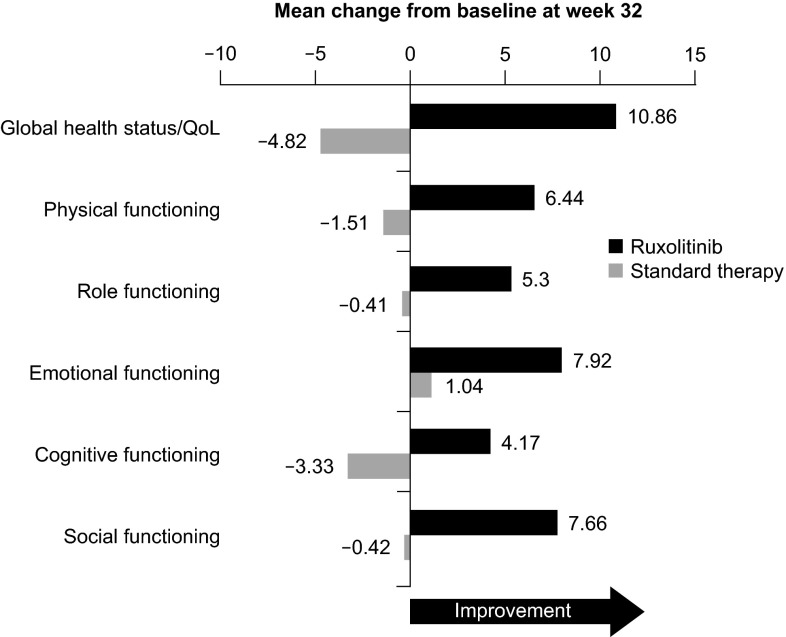
Mean change from baseline in EORTC-QLQ-C30 QoL and functioning scores at week 32 in patients with polycythemia vera in the RESPONSE study [98]. *EORTC*-*QLQ*-*C30* European Organization for Research and Treatment of Cancer Quality-of-Life questionnaire core model 30, *QoL* quality of life From [98]. Copyright © 2015 Massachusetts Medical Society. Reprinted with permission from Massachusetts Medical Society

The composite primary endpoint was achieved by 20.9 % of patients in the ruxolitinib arm compared with 0.9 % in the standard therapy arm (*P* < 0.001). Further analysis of the individual components of the primary endpoint showed that 60.0 % of patients in the ruxolitinib arm versus 19.6 % in the standard therapy arm had hematocrit control and 38.2 % in the ruxolitinib versus 0.9 % in the standard therapy arm (i.e., a single patient who received hydroxyurea) had a ≥35 % reduction in spleen volume from baseline to week 32. In addition, 49 % of patients receiving ruxolitinib compared with 5 % receiving standard therapy had a ≥50 % reduction in total symptom score, as assessed with a 14-item MPN Symptom Assessment Form patient diary. Median reductions in symptom score from baseline to week 32 for each of the 14 symptoms ranged from 37.1 % (numbness or tingling in hands or feet) to >90 % (itching, night sweats, sweating while awake, and early satiety). In contrast, standard therapy was associated with no or minimal symptom improvement or with symptom worsening (Fig. 4). The difference in efficacy between the two arms was reflected in the mean changes from baseline in EORTC-QLQ-C30 QoL and functioning scores, whereas ruxolitinib therapy resulted in overall improvement, standard therapy was associated with worsening (Fig. 5) [98]. Recent longer-term follow-up data suggest that the benefits of ruxolitinib therapy are durable. Among patients who achieved the composite primary endpoint, only one patient had lost response at the 80-week follow-up, and 82.7 % of patients were still receiving ruxolitinib after a median exposure of 111 weeks [99].

The superior benefit of ruxolitinib versus standard therapy was also reflected in the discontinuation rates for the two treatment arms. After a median treatment exposure of 81 weeks in the ruxolitinib arm, 15.5 % of patients had discontinued and none discontinued for protocol-defined lack of efficacy. In contrast, 96.4 % of patients randomized to standard therapy discontinued treatment or crossed over to ruxolitinib after a median treatment exposure of 34 weeks, and 87.5 % did so for lack of efficacy.

### Safety and tolerability

The hematologic safety profile in the RESPONSE study was similar for both treatment arms (Table 4) [98]. The most common grade 3 and 4 hematologic events were lymphopenia (ruxolitinib: 15.5 % and 0.9 %, respectively; standard therapy: 16.2 and 1.8 %, respectively). Two patients in the ruxolitinib arm (1.8 %) and no patients receiving standard therapy experienced grade 3 or 4 anemia. Grade 3 or 4 thrombocytopenia occurred in six (5.4 %) patients receiving ruxolitinib and four (3.6 %) patients receiving standard therapy.

**Table 4:** Hematologic adverse events^a^ from start of study drug to week 32 of the RESPONSE trial, regardless of relation to study drug [98]

Percent of patients with any grade event (percent with grade 3/grade 4 event)	Ruxolitinib (*n* = 110)	Standard therapy (*n* = 111)
Anemia	43.6 (0.9/0.9)	30.6 (0/0)
Thrombocytopenia	24.5 (4.5/0.9)	18.9 (2.7/0.9)
Lymphopenia	43.6 (15.5/0.9)	50.5 (16.2/1.8)
Leukopenia	9.1 (0.9/0)	12.6 (1.8/0)
Neutropenia	1.8 (0/0.9)	8.1 (0.9/0)

^a^New or worsening hematologic events based on laboratory values

Nonhematologic adverse events were generally grade 1 or 2 in both treatment arms [98]. Adverse events that were more common in the ruxolitinib versus the standard therapy arm (all-grade rates) included diarrhea (14.5 vs 7.2 %), muscle spasms (11.8 vs 4.5 %), and dyspnea (10.0 vs 1.8 %). In contrast, adverse events of pruritus, one of the most common symptoms of PV, occurred less frequently in the ruxolitinib versus the standard therapy arm (13.6 vs 22.5 %), likely reflecting the superior efficacy of ruxolitinib in symptom mitigation.

Within the 32-week study period, one patient treated with ruxolitinib versus six patients receiving standard therapy experienced thromboembolic events. Two patients died after crossover from standard therapy to ruxolitinib. The causes of death were central nervous system hemorrhage and multi-organ failure-associated hypovolemic shock and a sudden unexplained decrease in hemoglobin [98].

### Dosing

The recommended starting dose of ruxolitinib for the treatment of patients with PV is 10 mg twice daily. Because of the different hematologic characteristics of PV and advanced MF, ruxolitinib-induced anemia and thrombocytopenia are much less common in patients with PV than they are in patients with intermediate- or high-risk MF. In the ruxolitinib arm of the RESPONSE study, only 10 of 98 patients assessed at week 32 received doses that were lower than the starting dose, whereas 55 patients received higher doses (15, 20, or 25 mg twice daily) [98]. Dose reduction should be considered when a patient has a hemoglobin value in the range of 100 to <120 g/L and a platelet count of 75 to <100 × 10^9^/L. Dose reductions and interruptions are recommended if hemoglobin is >100 and >80 g/L, respectively, or platelet count is <75 × 10^9^ and <50 × 10^9^/L, respectively (for additional detail, see full prescribing information [20]).

## General safety considerations

Based on the pharmacokinetic profile of ruxolitinib, it is recommended that for concurrent administration with strong CYP3A4 inhibitors, the starting dose of ruxolitinib be reduced (Table 3) [20]. Patients already on a stable dose of ruxolitinib should also have their dose reduced if they begin taking a strong CYP3A4 inhibitor. Strong CYP3A4 inhibitors include but are not limited to boceprevir, clarithromycin, conivaptan, indinavir, itraconazole, ketoconazole, lopinavir, mibefradil, nefazodone, nelfinavir, posaconazole, ritonavir, saquinavir, telaprevir, telithromycin, and voriconazole. Because fluconazole is both a CYP3A4 and CYP2C9 inhibitor, it should not be taken at doses greater than 200 mg daily concomitantly with ruxolitinib. Furthermore, ingestion of grapefruit or grapefruit juice should be avoided in patients receiving ruxolitinib. Apart from grapefruit, ruxolitinib can be taken with or without food, as administration with food including high-fat, high-calorie meals versus administration without food met the bioequivalence criteria for bioavailability [20].

### Renal and hepatic impairment

Patients with renal or hepatic impairment exhibit altered pharmacokinetic properties of ruxolitinib and/or its metabolites [100]. Renal impairment had no effect on ruxolitinib pharmacokinetics but increased the exposure of ruxolitinib metabolites, with the greatest effect seen in patients with end-stage renal disease. Because these changes were associated with increased pharmacodynamic activity (inhibition of STAT3 phosphorylation) [100], dose reductions are recommended for patients with moderate or severe renal disease. Ruxolitinib is not recommended for patients with end-stage renal disease, unless they are on dialysis (Table 5) [20]. Hepatic impairment did not affect maximal serum concentration but increased exposure (by a factor of approximately 1.5–2) and decreased clearance, with no obvious quantitative relationship to the severity of impairment [100]. Accordingly, lower starting doses are recommended for all patients with MF or PV who have mild to severe hepatic impairment (Table 5) [20].

**Table 5:** Dosing of ruxolitinib in patients with renal or hepatic impairment [20]

Population	Platelet count	Ruxolitinib starting dose
Renal impairment		
MF: moderate (CrCl 30–59 mL/min) or severe (CrCl 15–29 mL/min) impairment	>150 × 10^9^/L	No dose modification
	100 × 10^9^ to 150 × 10^9^/L	10 mg twice daily
	50 × 10^9^ to <100 × 10^9^/L	5 mg daily
	<50 × 10^9^/L	Not recommended
PV: moderate or severe impairment	Any	5 mg twice daily
MF or PV: end-stage renal disease (CrCl < 15 mL/min) on dialysis	>200 × 10^9^/L	20 mg once after dialysis session
	100 × 10^9^ to 200 × 10^9^/L	15 mg once after dialysis session
MF or PV: end-stage renal disease (CrCl < 15 mL/min) not requiring dialysis	Any	Not recommended
Hepatic impairment (mild to severe)		
MF	>150 × 10^9^/L	No dose modification
	100 × 10^9^ to 150 × 10^9^/L	10 mg twice daily
	50 × 10^9^ to <100 × 10^9^/L	5 mg daily
	<50 × 10^9^/L	Not recommended
PV	Any	5 mg twice daily

*CrCl* creatinine clearance, *MF* myelofibrosis, *PV* polycythemia vera

### Risk of infection

In vitro and ex vivo evidence of inhibition of dendritic cell function and downregulation of T regulatory cells with ruxolitinib [101–103] suggests that ruxolitinib may have immunosuppressive activity. However, ruxolitinib does not appear to inhibit the formation of T cells, as evidenced by the successful immune reconstitution experienced by patients who received ruxolitinib and stem cell transplantation [104]. Nonetheless, isolated cases of serious infections have been noted in patients with MF who were treated with ruxolitinib in clinical practice, including progressive multifocal leukoencephalopathy [105], increase in hepatitis B viral titers [106], reactivation of herpes simplex virus [107], and disseminated tuberculosis [108]. Furthermore, herpes zoster and urinary tract infections occurred in patients treated with ruxolitinib in phase 3 trials in MF and PV [20]. Because of the potential for bacterial, fungal, or viral infections to occur or be reactivated in patients receiving ruxolitinib, special care should be taken when treating patients with MF who have an already compromised immune system due to other underlying medical problems or comorbidities, or those who have a history of atypical infections. Patients should be observed for signs of infection so that proper treatment can begin promptly, and ruxolitinib should not be started until serious infections have resolved. Patients at risk of tuberculosis (i.e., patients who have a history of tuberculosis, have lived in or traveled to countries with a high prevalence of tuberculosis, or have had close contact with a person with active tuberculosis) should be tested for latent infection prior to starting ruxolitinib. Those who test positive should consult with their physician regarding the use of ruxolitinib based on their overall benefit-risk ratio [20].

### Cardiac safety

The effect of single-dose ruxolitinib (25 and 200 mg) on the QTc interval was evaluated in a randomized, placebo- and active-controlled (moxifloxacin 400 mg) four-period crossover thorough QT study in 47 healthy human participants [109]. In this study, which had a demonstrated ability to detect small effects, the upper bound of the one-sided 95 % CI for the largest placebo-adjusted, baseline-corrected QTc (Fridericia correction method) was <10 ms, which is below the level of regulatory concern. The dose of 200 mg is adequate to represent the high-exposure clinical scenario [20].

## Future directions

Although ruxolitinib has proven to provide unprecedented clinical benefits for patients with MF in terms of spleen size reduction, symptom alleviation, and improvements in measures of quality of life, it has also become clear in recent years that single target-directed therapies are unlikely to provide high levels of responses against the underlying clonal neoplasm in a majority of patients with advanced disease. Despite the fact that ruxolitinib targets the most critical pathway driving neoplastic proliferation in MPNs (i.e., the JAK2-STAT pathway), its capacity to elicit significant molecular responses (i.e., allele burden reductions) and/or bone marrow morphologic responses such as reversal of fibrosis in MF appears to be limited to a minority of patients and are generally only achieved with sustained long-term therapy [77, 78]. It has been hypothesized that inflammation-imprinting of the bone marrow stroma in PMF by mutant clonal hematopoietic cells eventually results in changes in the stroma that protect the mutant clones against JAK2 inhibitor therapy [110]. This may explain, at least in part, why ruxolitinib is highly effective in providing rapid relief from MF-related symptoms, which to a large extent are attributed to excess inflammatory cytokine expression, without having a major effect on clonal proliferation in patients with advanced MF [21, 61]. Similarly, the survival benefit observed with ruxolitinib in these patients is likely the result of effective disruption of the vicious cycle of self-sustaining inflammation and the consequent improvement of overall clinical status rather than of the moderate effects on myeloproliferation. Further improvement of survival in patients with MF may come from combination therapies of ruxolitinib with agents that target complementary pathogenic pathways, inhibit aberrant epigenetic modification (e.g., histone deacetylation), and help to reverse fibrosis in the bone marrow; these novel therapeutic approaches are currently being evaluated in numerous early-phase clinical trials [111].

## Conclusions

Results of phase III clinical trials have demonstrated that the JAK1 and JAK2 inhibitor ruxolitinib provides significant clinical benefits for patients with advanced MF and patients with PV who have had inadequate response to or are intolerant of hydroxyurea, including but not limited to reduction of splenomegaly and symptom burden in MF and PV and hematocrit control in PV. These findings are consistent with the critical involvement of dysregulated JAK–STAT in the pathobiology of both diseases, irrespective of the type of pathogenic mutations. Although ruxolitinib is not curative, current evidence suggests that it improves overall QoL, has prolonged survival in controlled clinical trials of patients with MF, and in some cases may lead to complete resolution of bone marrow fibrosis. Ruxolitinib is generally well tolerated but its myelosuppressive activity may lead to de novo or worsening cytopenias, particularly in patients with advanced MF, who often have disease-related anemia and/or low platelet counts. Careful monitoring of complete blood counts and prompt dose adjustments, particularly during the first 3 months of therapy, are essential tools of patient management to avoid dose-dependent cytopenias and ensure sustained, uninterrupted therapy, which in turn is required to maintain treatment responses.

In summary, ruxolitinib is appropriate for the treatment of splenomegaly and MF-related symptoms in patients with intermediate- or high-risk MF who have a platelet count of ≥50 × 10^9^/L and do not qualify or opt for allogeneic hematologic stem cell transplantation. Ruxolitinib-mediated reductions in spleen size and symptom burden, and even improvement of survival appear not to be dependent on the patient’s mutation profile [75]. However, the ultimate treatment decision should be based for each patient on the totality of patient- and disease-specific criteria, including age, performance-status, risk category, mutation profile, degree of symptoms and splenomegaly, and presence of disease-related cytopenias. In PV, ruxolitinib should be considered as a treatment option for patients with uncontrolled hematocrit who are refractory to or do not tolerate hydroxyurea, particularly if the patient also has splenomegaly and/or PV-related symptoms.
